# Data-driven optimization of preschoolers’ hemodynamic response in a VR setup: advancing analytic methods for children’s fNIRS naturalistic data with the AICopt method

**DOI:** 10.1117/1.NPh.13.2.025004

**Published:** 2026-06-08

**Authors:** Letizia Contini, Rebecca Re, Paola Pinti, Chiara Bulgarelli

**Affiliations:** a Politecnico di Milano, Dipartimento di Fisica, Milan, Italy; b Istituto di Fotonica e Nanotecnologie, Consiglio Nazionale delle Ricerche, Milan, Italy; c University of London, Centre for Brain and Cognitive Development, Birkbeck, United Kingdom; d University College London, Department of Medical Physics and Biomedical Engineering, London, United Kingdom

**Keywords:** functional near-infrared spectroscopy, naturalistic neuroscience, immersive virtual reality, preschoolers’, functional near-infrared spectroscopy data, data-driven optimization, hemodynamic response function, general linear model.

## Abstract

**Significance:**

Naturalistic fNIRS data acquired on children enable studying real-world behaviors but challenge standard analysis methods such as block averaging and general linear model (GLM). In naturalistic paradigms, events often overlap, whereas children’s hemodynamic responses generally deviate from the adult canonical model, possibly leading to responses’ misattribution and low sensitivity.

**Aim:**

We aim to reduce the risk of misattributing neural responses to stimuli by refining the shape and timing of the hemodynamic response function (HRF) for each brain region and event type in a data-driven framework, addressing cases where overlapping responses lead to neural responses being mistakenly assigned to the wrong stimulus, distorting results, and leading to misleading conclusions.

**Approach:**

We introduce a data-driven HRF optimization procedure (AICopt) that enables GLM-based analyses when the HRF is unknown. We evaluated the AICopt approach in 40 preschoolers (3 to 5 years) within a virtual-reality paradigm, featuring emotionally relevant and neutral events followed immediately by choices, without fixed inter-trial baselines. Then, we compare its performance with what is obtained using the block-averaging method and canonical HRF model-based GLM analysis.

**Results:**

AICopt yielded activation patterns that converged with block-averaging results for events while avoiding likely spurious choice-related activations seen with the canonical GLM. Overall, the use of data-driven HRFs improved sensitivity and reduced misattribution relative to the fixed canonical HRF in this overlapping-event design.

**Conclusions:**

Our results suggest that data-driven HRF modeling is a necessary step when analyzing fNIRS data from atypical populations such as young children, particularly in studies employing naturalistic setups. The presented AICopt method represents a possible approach to adapt GLM analyses to overlapping events and diverse populations, improving accuracy and interpretability of the obtained activation maps, and offering a reusable workflow for child fNIRS datasets collected in nonstandard setups.

## 1 Introduction

The developmental neuroscience community has recently acknowledged the importance of using more naturalistic testing setups to assess children’s development.^1^ Although decades of traditional standardized lab assessments have greatly contributed to the understanding of the developing brain and behavior, designing more naturalistic tasks is crucial for exploring behaviors in conditions that are not suitable for traditional lab set-ups, such as group interactions and situations that involve movements, or ages that generally struggle to comply with strict lab rules, such as preschoolers and young children.

The advancement of wearable neurotechnologies has allowed the field to take its first steps toward a more naturalistic approach to developmental neuroscience. Within this context, functional near-infrared spectroscopy (fNIRS) is a valuable tool to be used outside conventional settings. By leveraging the different absorption properties of oxygenated and deoxygenated blood, fNIRS shines weak near-infrared light through the participant’s scalp to measure changes in hemoglobin concentration in the cerebral cortex as a proxy for brain activation.^2^ Thanks to its portability, flexibility, and high tolerance by young participants, fNIRS has seen widespread use in developmental neuroscience, as evidenced by the significant increase in publications featuring developmental sample studies in recent years.^3^ Recently, most fNIRS companies have focused their resources on making fNIRS wearable by compacting the system into small boxes or backpacks, addressing the need to bring this tool outside the lab. The lightweight nature of these systems and their robustness to motion make fNIRS particularly well suited for its use with young children in real-world studies, where children can move freely and interact with the environment while their brain activity is measured.^4^ For example, our team has successfully pioneered the use of wearable fNIRS within a virtual-reality (VR) setup with toddlers, considered a valid mid-point between the unpredictable real world and a tightly controlled lab-set up. In a first study, we validated this combined fNIRS/VR setup to assess preschoolers’ social preferences by exposing 3-to-5-year-olds to virtual agents of different ages and genders. Although this allowed us to estimate functional connectivity in freely moving toddlers for the first time and identify different brain networks as participants interacted with different social partners, we acknowledge that the task design (i.e., asking participants to play a simple bubble-popping game with the virtual agents for a few minutes) was basic and possibly not suitable for assessing more sophisticated social skills.^5^ A second study aimed to investigate the effect of physiological contamination in the preschoolers’ fNIRS signal within the VR environment, testing children with a virtual-reality version of a classic go/no-go task.^6^ Although this was novel for the analysis of naturalistic developmental fNIRS data, the task used (i.e., popping only blue but not red bubbles while in a virtual playground) mimicked the timing of a standard block design task, with alternating conditions and rest periods. However, it is not always possible to confine the assessment of psychological domains within such a rigid temporal structure and, if we want to explore more complex cognitive functions, we need to move beyond standard and rigid block designs. The main advantage of designing realistic tasks is to overcome the limitations of standard assessments, such as habituation to the same set of stimuli through the task or impoverished behavioral and neural responses that may not be representative of real-world situations. However, this is not without challenges. In fact, neuroimaging tasks are designed based on the features of neurotechnology used and the characteristics of the hemodynamic response (HR) that we aim to capture with the task. The HR time-to-peak is ∼5  s in adults^7^ but typically takes up to 8 to 10 s in infants and toddlers.^8^ As a result, task conditions are often designed to last 8 to 12 s, typically including a baseline or a rest condition to allow the HR to return to baseline before the next trial. In addition, the HR signal-to-noise ratio is significantly lower compared with other aspects of the fNIRS signal, such as background physiological changes, motion artifacts, and measurement noise.^9^ As a result, researchers design multiple repetitions of the same stimuli to increase the HR signal-to-noise ratio. As when testing infants or young children, some trials may be excluded due to looking time or fussiness, which affects the number of trials we can average from the same condition; we often expose our young participants to more trials per condition than the minimum number required for inclusion in the final analyses. However, although developmental scientists have successfully been able to design traditional tasks that meet the mentioned HR features, it is obvious that real-world events do not occur in repeated 10-s segments.^4^ Moreover, finding periods of the appropriate baseline is challenging when testing outside the lab. It thus becomes of critical importance to advance the analysis of fNIRS data recorded in children in more unstructured contexts to deal with unstructured designs with overlapping events.

In standard neurodevelopmental studies, the HR response is usually recovered using an averaging approach, i.e., averaging segments of the signal around the stimulus onset to determine a specific brain region average response to a certain condition; parameters reflecting the amplitude of the response (e.g., mean, peak) are then extracted and used for statistical testing. However, this method is not feasible when multiple events occur close to each other, and therefore, the HRs may overlap. On the other hand, generalized linear model (GLM) analyses are better suited for designs with closely spaced or overlapping events and are generally able to disentangle overlapping responses.^10^ However, the GLM often relies on the assumption of a hemodynamic response function (HRF), which is well characterized for healthy adults (i.e., the canonical double-gamma HRF), whereas no standardized or widely accepted HRF model is currently available for young pediatric populations or for other cohorts such as clinical groups.^11^ Although general features of developmental HRFs have been reported (e.g., delayed and reduced responses compared to adults), age-specific HRF models, particularly for toddlers and preschool-aged children, remain largely undefined.^8^^,^^11^ Although adjusted HRF models have been proposed for neonates and infants, these estimates are unlikely to generalize to preschool-aged children, as neurovascular coupling undergoes substantial maturation across the first years of life. The morphology and timing of the hemodynamic response are known to change systematically with age, rendering direct extrapolation from infant HRF models to older pediatric populations unreliable.^11^ For readers who are not familiar with GLM analysis, please refer to Huppert et al.,^10^ for an introduction to its application in fNIRS.

Here, we aim to fill this gap, and we propose a novel data-driven HRF optimization algorithm that builds on the widely used double-gamma canonical model. Unlike classical methods that rely on the canonical HRF to model the HR, our approach allows for the extraction of the optimal HRF model from the data itself, making it particularly well suited for enabling the use of GLM analyses in event-related designs with closely spaced or overlapping events, and making them reliable when the true HRF is not known a priori (e.g., in children and/or clinical cohorts). Similar strategies have previously been employed in infant neuroimaging studies to improve GLM modeling by adjusting the parameters describing the features of the canonical HRF to better reflect the temporal dynamics observed in young populations.^12^ In this study, the grand-averaged fNIRS signals were iteratively fitted with multiple HRF models, each varying key temporal parameters, to identify the best-fitting shape for subsequent analyses. Building upon this idea, the proposed Akaike information criterion-driven optimization (AICopt) method first performs a block averaging of the fNIRS data to obtain an initial estimate of the HR for each brain region and condition. Then, multiple GLMs are iteratively applied to these block-averaged datasets, each assuming a different variation of the double-gamma HRF. Finally, the Akaike information criterion (AIC),^13^ a metric used to quantify how well a model fits a set of data based on its log-likelihood value, is used to evaluate each model fit and identify the one providing the best explanatory power.^14^ The associated optimal HRF model can subsequently be used for further analyses, such as GLM-based activation mapping. A key advantage of this method in studies involving events in close temporal proximity, as it is likely to happen in real-world assessments, is its ability to accommodate the nonlinear superimposition of multiple HRs. In addition, the proposed method avoids reliance on standard HRF assumptions, which may not be optimal for young populations, thereby enhancing sensitivity to task-related responses.

The overall aim of this work is to test for the first time the proposed AICopt method on an fNIRS dataset acquired in a nontraditional testing setup on preschool-aged children and to explore potential errors or misinterpretations that may arise when the HR or task structure is not appropriately modeled by directly comparing AICopt-based GLM results with a canonical HRF-based GLM. This enables us to assess whether reliance on the fixed canonical HRF leads to significant biases relative to using a data-driven HRF. To this end, we will use fNIRS data acquired on N=40 3-to-5-year-old participants freely moving in a VR environment as part of a study investigating neural markers of empathy in the Birkbeck ToddlerLab Cave Automatic Virtual Environment (CAVE) facility. In this study, preschoolers interacted with virtual human-like toddlers and were exposed to a set of emotionally relevant events (aimed to elicit empathy) and emotionally neutral ones. Soon after each event, participants were asked to choose how to react to the event by selecting between two possible actions. To preserve the realistic features of the scenes, there was no standard/typical baseline between events or between the events and the subsequent action choices, which posed a risk that the preschoolers’ HR would not return to baseline before the next event, introducing analytical challenges. Therefore, due to the temporal proximity of the stimuli used in our protocol, conventional analysis methods such as block-averaged and standard GLM analyses are inadequate for recovering the neural responses.

In this work, we aim to reduce the risk of misattributing neural responses to events by refining the shape and timing of the HRF for each brain region and event type, addressing cases where a delayed HR peak is mistakenly assigned to the wrong stimulus, distorting results, and leading to misleading conclusions. To this end, this work compared the performance of these three analytical methods applied to the same dataset:

1. Block averaging, the conventional approach in fNIRS research on children, which avoids assumptions about HR shape and can accommodate variability in HRs^15^ but is susceptible to overlap effects, potentially leading to overestimation of activation.^8^
2. Canonical GLM, the standard method in adult fNIRS studies, which models task-related activation using a fixed double-gamma HRF derived from adult populations but does not adapt to local differences and may misattribute neural responses in nonstandard populations.^16^
3. AICopt-based GLM, a novel data-driven approach that estimates optimized HRF for each brain region and condition based on the actual dataset, allowing for more flexible modeling of response shapes and timings.

Validating this method will provide a novel data-analysis framework that others in the field can adopt for making GLM-based deconvolution more reliable in real-world fNIRS datasets, particularly in pediatric and clinical contexts.

## 2 Methods

### 2.1 Participants

Forty 3-to-5-year-olds were enrolled in the study (19 males, age mean±SD=4.52±0.81  years). Six additional participants were recruited but not included in the final analyses because: (i) they refused to wear the fNIRS hat and/or do the task (four participants); (ii) technical issue (1 participant); (iii) excessive motion and noise in the data (1 participant). All included participants were born full-term, healthy, and with normal birth weight. Participants were excluded from recruitment if they had a known significant neurodevelopmental condition or a medical condition that was likely to impact brain development or impede the child’s ability to participate in this study. Written informed consent was obtained from the preschooler’s caregiver prior to the start of the experiment. Ethical approval for this study was given by the Ethics Committee of the Department of Psychological Sciences, Birkbeck, University of London (No. 2122056).

### 2.2 Apparatus, Stimuli, and Procedure

**Fig. 1:**
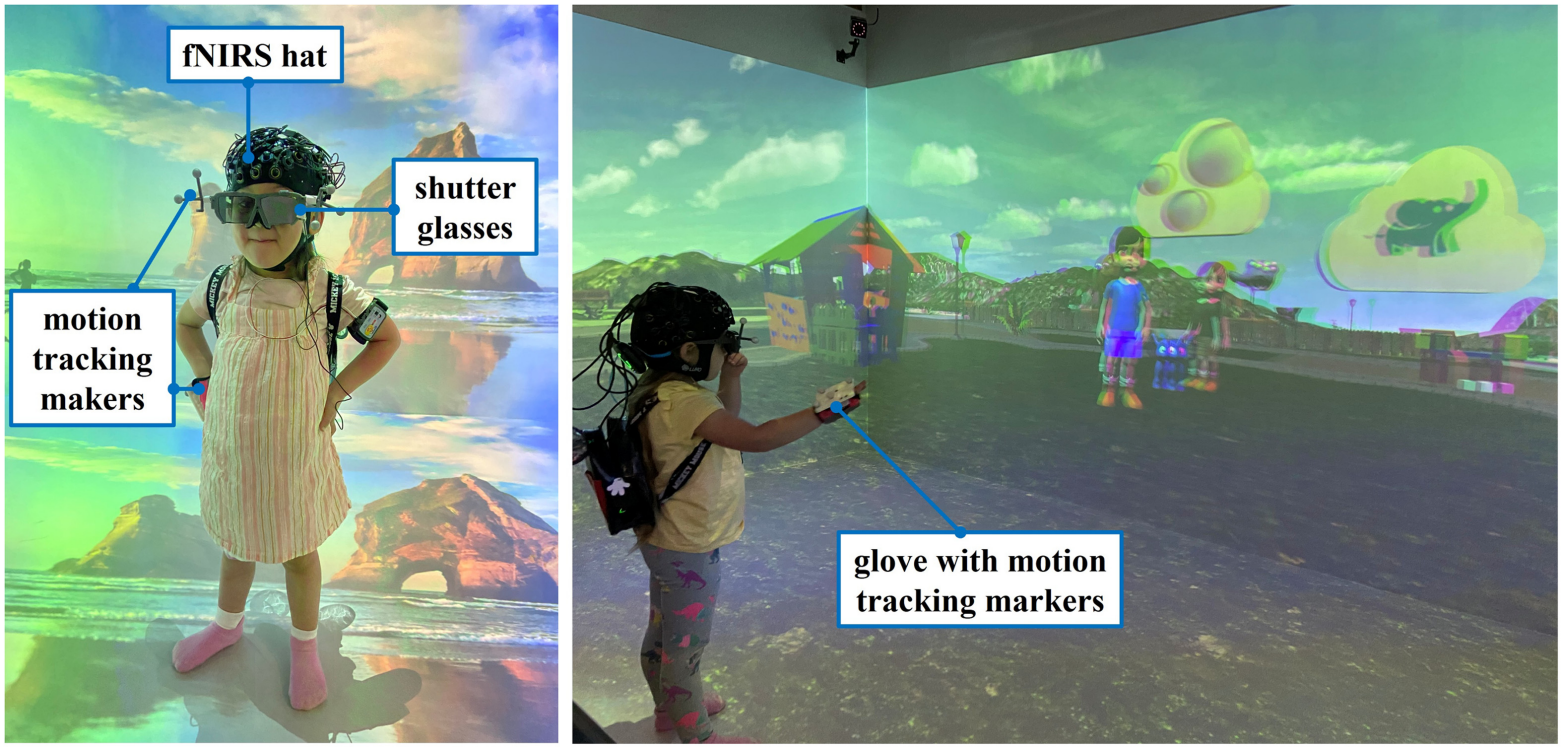
Cave automatic virtual environment (CAVE) at the Birkbeck ToddlerLab, and equipment worn by children during the experiment.

The experiment took place in the immersive Cave Automatic Virtual Environment at the Birkbeck ToddlerLab (Fig. 1, for more technical information regarding the CAVE see Bulgarelli et al.,^5^).

At the beginning of the experiment, the participant was made familiar with the CAVE set-up and the equipment, presented in Fig. 1. First, the fNIRS cap and custom-built child-sized LCD (i.e., liquid crystal display) shutter glasses that enabled active-stereo viewing to increase the immersiveness of the experience in the CAVE were fitted on the participant’s head. Six passive markers for head tracking were attached to the frame glasses to reorient and rotate the virtual scenes according to the participant’s head position. Motion tracking markers were also attached onto a cyclist glove to allow participants to interact with virtual objects in the scene (the same glove was present in the virtual scenario to represent the participant’s hand, and it moved in sync with the participant’s real hand). The VR task was implemented using Unity (v 2019.4.18f1) in conjunction with a CAVE-specific plugin (getReal3D, Mechdyne) to allow every image to be synchronized across all the projectors. The task was set in a virtual playground as children of this age are familiar with such environments from their daily life experiences, and it has been used previously in another study from our team.^6^ Initially, the participants were invited to explore the playground and test the correspondence between their hand with the glove and the hand in the virtual playground.

Once the participants were comfortable navigating the virtual playground and moving their hands, they were exposed to some practice trials, in which they were presented with two clouds, one containing an orange star and the other a green star. In the first practice trial, a native English-speaking voice said: “By touching the cloud, you make things happen in the game.” The experimenter then asked the participant to touch either the cloud with the orange star or the one with the green star, and each time the child was successful, golden sparkles appeared in the background. This was crucial for the preschoolers to understand that by touching different clouds in the game with their gloved hand, they could interact with the virtual environment and that they could touch only one cloud at the time. Practice trials were repeated until the participants were confident in touching the clouds with their gloved hand.

**Fig. 2:**
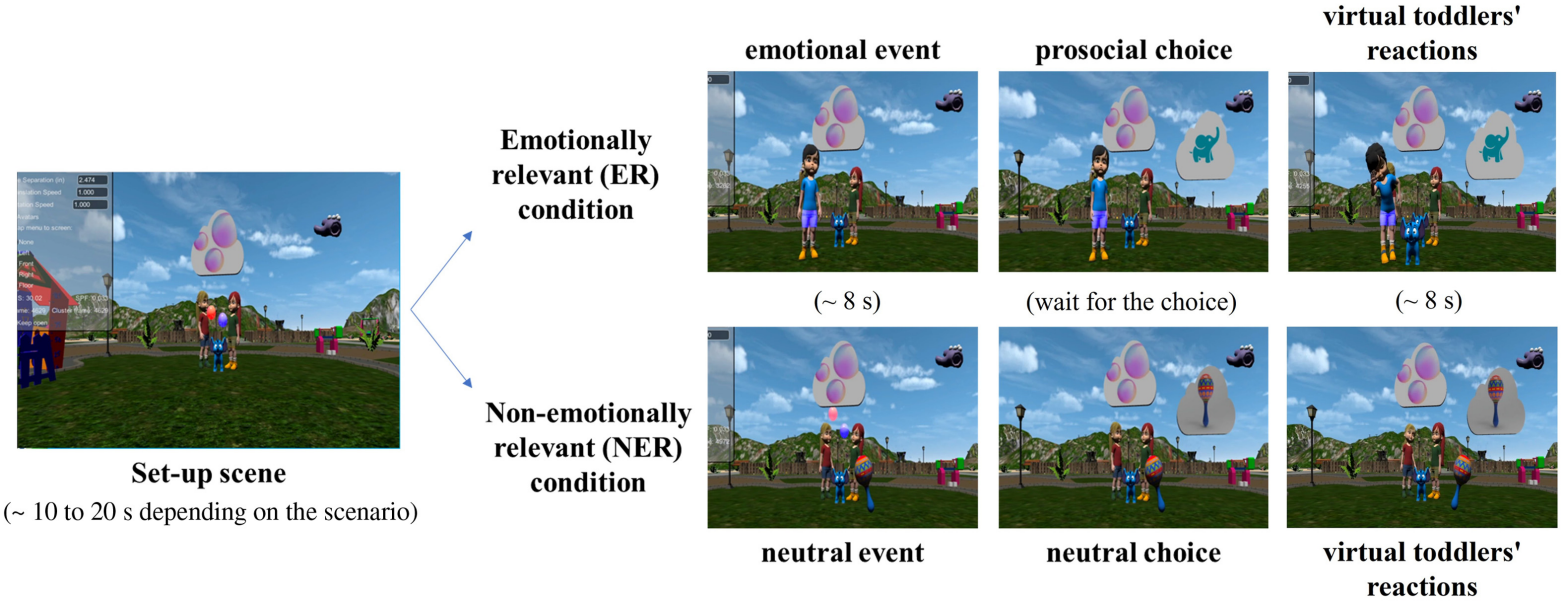
Depiction of the scene seen by the toddlers for an example of ER and NER scenarios.

After the practice trails were completed, participants were exposed to three emotionally relevant (ER) scenarios and three nonemotionally relevant (NER) scenarios in this order: NER-ER-ER-NER-ER-NER. If the child was still fine with the equipment on, the first three or four trials were then repeated. Each scenario had a setup scene that could vary in length (∼10 to 20 s, depending on the scenario) but always featured virtual toddlers and a cloud, which the participant was encouraged to touch to interact with. As soon as a new scenario began, a native English-speaking voice instructed the participant on what would happen if they touched the cloud, and the child was expected to touch the cloud in the scene to interact with the environment. Afterward, either an ER or NER event occurred in the scene (event). Approximately 8 s later, a second cloud appeared next to the first one, and a background voice instructed the participant on what would happen if they touched one cloud or the other (choice). The participant’s choice was then recorded, the scene updated, and the selected outcome was displayed for ∼8  s. A new scenario began after a few seconds. Although the overall timing of each scenario depended on the participant’s interaction with the VR environment and on the specific scene, minimum durations for each phase were forced to ensure a consistent temporal structure across subjects. Each ER scenario was paired with a corresponding NER scenario, meaning the set-up was the same, and the participant did not know the emotional valence of what would happen afterward. All scenarios took place in the aforementioned virtual playground, which remained the same throughout and was always visible between scenarios. Each scenario featured virtual toddler girls, as our previous work showed that 3-to-5-year-olds, regardless of their gender, preferred interacting with this virtual partner.^5^ See Table 1 for a full description of the ER and NER scenarios and Fig. 2 for a depiction of the scenes seen by the toddlers for one example of ER and NER scenario.

**Table 1:** Description of the ER and NER scenarios that participants are exposed to.

No of scenario	Condition	Set-up	Event	Actions	
				Option 1	Option 2
1	NER 1	Two virtual toddlers are in front of a blue elephant bubble machine. By touching the cloud, the participant makes the two virtual toddlers pop bubbles emerging from the machine.	A maraca appears next to the toddler avatars.	The virtual toddlers listen to some music.	The virtual toddlers keep playing popping bubbles.
2	ER 1	Two virtual toddlers are on a trampoline. By touching the cloud, the participant makes the virtual toddlers jumping.	One virtual toddler fell over.	The participant helps the virtual toddler on the ground to stand up.	The virtual toddler on the trampoline keeps jumping.
3	ER 2	Three virtual toddlers are in front of a plate with four biscuits, and there is some mud on the grass. By touching the cloud, the participant gives the three virtual toddlers a biscuit each, and they are told the fourth biscuit is for themselves.	One virtual toddler drops her biscuit in the mud.	The participant shares their biscuit with the virtual toddler who dropped hers.	The participant keeps their biscuit.
4	NER 2	Two virtual toddlers are on a trampoline. By touching the cloud, the participant makes the virtual toddlers jump.	A book appears next to the trampoline.	The virtual toddlers listen to a story	The virtual toddlers on the trampoline keep jumping.
5	ER 3	Two virtual toddlers are in front of a blue elephant bubble machine. By touching the cloud, the participant makes the two virtual toddlers pop bubbles emerging from the machine.	A third toddler avatar comes in and asks to play, but one of the two others says no.	The participant gives another bubble machine to the virtual toddler excluded.	The virtual toddlers keep playing popping bubbles.
6	NER 3	Three virtual toddlers are in front of a plate with four biscuits, and there is some mud on the grass. By touching the cloud, the participant gives the three virtual toddlers a biscuit each, and they are told the fourth biscuit is for themselves.	Three lollipops appear next to the three biscuits.	The participant gives a biscuit to each virtual toddler.	The participant gives a lollipop to each virtual toddler.

### 2.3 FNIRS Data Acquisition

fNIRS recordings were acquired using two portable Brite MKII systems (Artinis Medical Systems BV, Netherlands) operating at two continuous near-infrared wavelengths: 763 and 841 nm, with a sampling rate of 25 Hz. The fNIRS systems were used to monitor relative concentration changes in oxygenated (${\mathrm{HbO}}_{2}$) and deoxygenated hemoglobin (HHb). Each device was equipped with 18 optodes (10 light sources and 8 detectors), which were embedded in a soft neoprene cap (EasyCap). The cap assured a secure and repeatable placement of each optode on the participant’s head. The two devices were positioned to cover both the frontal lobe (device #1) and temporo-parietal regions (device #2) bilaterally. A total of 48 channels were used: 24 over the dorsal and medial frontal regions and 12 over each temporo-parietal region. A source-detector (SD) distance of about 25 mm was set for most channels, excluding two short-separation channels (SSC), with a 10 mm SD separation, positioned on the frontal lobe (channel 12) and the left temporal lobe (channel 37). This distance corresponds to the minimum source-detector separation allowed by the system used in this study and has been previously validated for short-channel regression in toddlers and preschoolers using age-appropriate head models by Pinti et al.^6^ The signal recorded from the two SSCs was used to account for extracerebral and systemic physiology-induced changes of ${\mathrm{HbO}}_{2}$ and HHb during data processing (see paragraph Sec. 2.5). Both devices were equipped with built-in accelerometers to measure the participant’s motion.

**Fig. 3:**
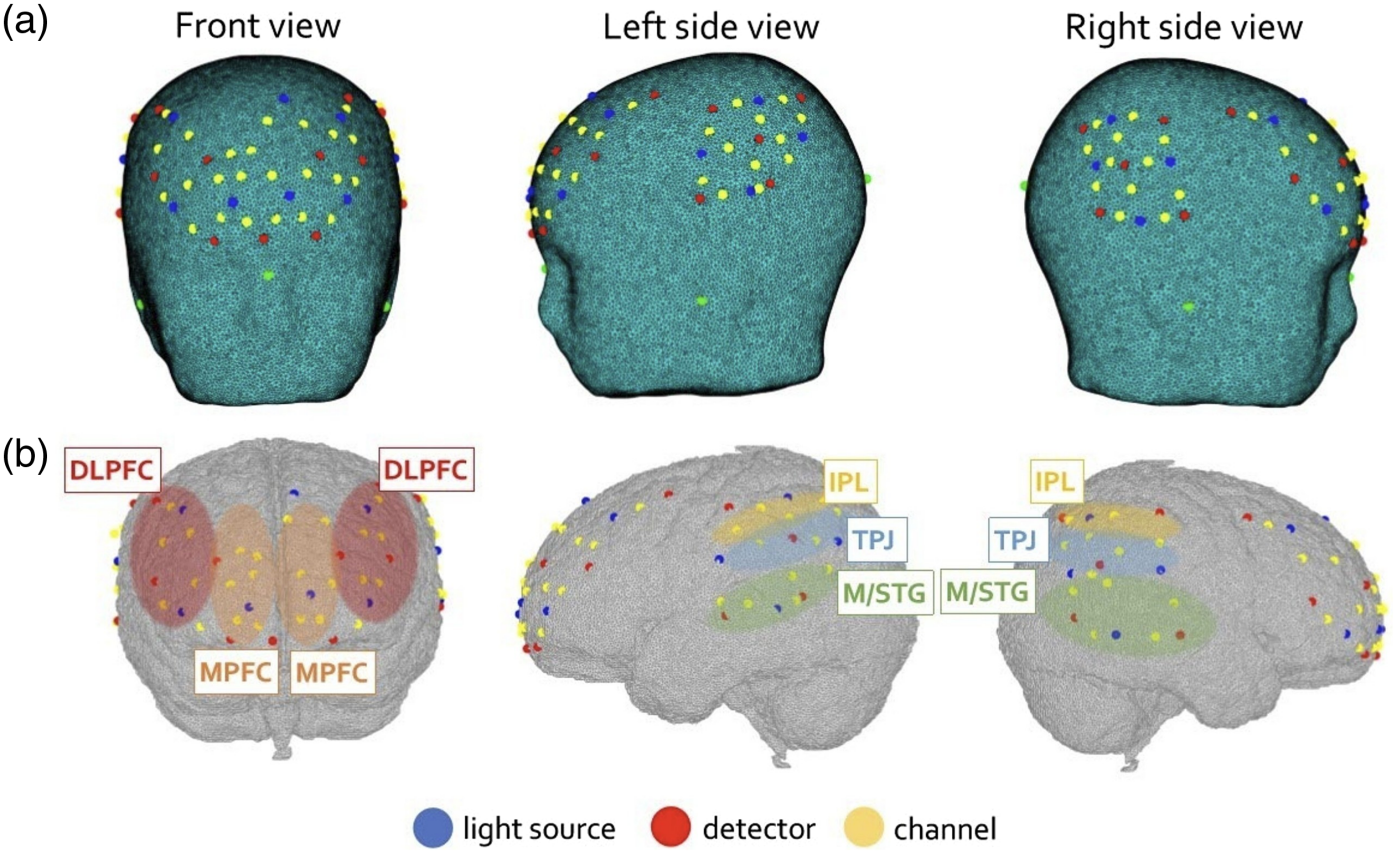
(a) Representation of the channels on a 5-year-old multilayer head model mesh; (b) schematic representation of the channels overlapped onto the gray matter mesh extracted from the multilayer volumetric template model. ROIs are highlighted: orange represents MPFC, red represents DLPFC, green represents M/STG, blue represents TPJ, yellow represents IPL. Figure reproduced with permission from Bulgarelli et al.^5^

To minimize the weight on the child’s head, only one unit was mounted at the back of the cap to capture head movement, whereas the other was carried in a backpack worn by the participant. All channels were coregistered onto an age-appropriate MRI template to create 10 regions of interest (ROI, left and right medial prefrontal cortex, MPFC; left and right dorsolateral prefrontal cortex, DLPFC; left and right temporo-parietal junction, TPJ; left and right middle and superior temporal gyrus, M/STG; left and right inferior parietal lobule, IPL), following a previous work using the same systems and the same array design on a 3-to-5-year-olds sample (see Fig. 3 and Table 2, reproduced from Bulgarelli et al.^5^).

**Table 2:** Co-registration of each channel of the fNIRS array. The table shows anatomical labels (LPBA40 atlas) and ROI associated with each channel. Table reproduced from Bulgarelli et al.^5^

Frontal lobe			Temporo-parietal lobe		
Channel no.	LPBA label	ROI	Channel no.	LPBA label	ROI
1	Right inferior frontal gyrus	/	25	Right middle temporal gyrus	Right M/STG
2	Right middle frontal gyrus	Right DLPFC	26	Right middle temporal gyrus	Right M/STG
3	Right middle frontal gyrus	Right DLPFC	27	Right middle temporal gyrus	Right M/STG
4	Right middle frontal gyrus	Right MPFC	28	Right angular gyrus	Right TPJ
5	Right superior frontal gyrus	Right MPFC	29	Right angular gyrus	Right TPJ
6	Right middle frontal gyrus	Right MPFC	30	Right middle temporal gyrus	Right M/STG
7	Right superior frontal gyrus	Right MPFC	31	Right superior temporal gyrus	Right M/STG
8	Right middle frontal gyrus	Right DLPFC	32	Right supramarginal gyrus	Right IPL
9	Right middle frontal gyrus	Right DLPFC	33	Right superior temporal gyrus	Right TPJ
10	Right middle frontal gyrus	Right DLPFC	34	Right angular gyrus	Right TPJ
11	Right superior frontal gyrus	Right MPFC	35	Right angular gyrus	Right IPL
12 (SSC)	Right superior frontal gyrus	/	36	Right angular gyrus	Right IPL
13	Left superior frontal gyrus	Left MPFC	37 (SSC)	Left middle temporal gyrus	/
14	Left middle frontal gyrus	Left MPFC	38	Left middle temporal gyrus	Left M/STG
15	Left superior frontal gyrus	Left MPFC	39	Left middle temporal gyrus	Left M/STG
16	Left middle frontal gyrus	Left MPFC	40	Left angular gyrus	Left TPJ
17	Left middle frontal gyrus	Left MPFC	41	Left angular gyrus	Left TPJ
18	Left middle frontal gyrus	Left MPFC	42	Left angular gyrus	Left M/STG
19	Left middle frontal gyrus	Left DLPFC	43	Left supramarginal gyrus	Left TPJ
20	Left middle frontal gyrus	Left DLPFC	44	Left supramarginal gyrus	Left IPL
21	Left middle frontal gyrus	Left DLPFC	45	Left superior temporal gyrus	Left M/STG
22	Left inferior frontal gyrus	/	46	Left angular gyrus	Left TPJ
23	Left middle frontal gyrus	Left DLPFC	47	Left angular gyrus	Left IPL
24	Left middle frontal gyrus	Left DLPFC	48	Left supramarginal gyrus	Left IPL

### 2.4 FNIRS Data Processing

Data analyses were conducted using in-house code developed in MATLAB (MathWorks, Natick, Massachusetts, United States), following steps largely based on our previous works published with the same fNIRS/VR setup.^5^^,^^6^ Raw intensity data were converted to optical density (*hmrIntensity2OD.m* function from Homer2 tool).^10^ Hereafter, motion artifacts were corrected using wavelet (iqr = 0.8, *hmrMotionCorrectWavelet.m* function from Homer2). Low-quality channels, based on physiological indicators of quality, were pruned using QT-NIRS.^17^ For each participant, data quality was assessed channel-by-channel in nonoverlapping 3-s windows based on the Scalp Coupling Index (SCI) and the peak spectral power (PSP) (threshold SCI = 0.60, threshold PSP = 0.06, empirically defined).^17^ Channels that had both SCI and PSP below threshold for more than 60% of the windows were excluded from further pre-processing steps. Hereafter, the surviving channels underwent visual inspection, and channels with clear signs of noise or saturation were additionally removed. Optical density data were then bandpass filtered (0.01 to 0.5 Hz) (*hmrBandpassFilt.m* function from Homer2) and converted to relative concentrations of hemoglobin using the modified Beer–Lambert law (*hmrOD2Conc.m* function from Homer2, DPF = 5.4, 4.6).^18^ Hereafter, channels that survived pre-processing were grouped according to the 10 ROIs defined above, based on Bulgarelli et al.,^5^ and the corresponding fNIRS signals were obtained by averaging the time series of all channels belonging to each ROI. These ROI-level signals were used for the following analysis.

### 2.5 FNIRS Data Analyses

As we aimed to compare the performance of the AICopt method with the two conventional methods in developmental fNIRS studies, data were analyzed using: (i) a block averaging, (ii) a canonical GLM assuming the canonical double-gamma HRF, and (iii) a GLM using the optimized HRF obtained using the AICopt method:

#### 2.5.1 Block averaging

The hemodynamic responses are averaged across all trials of the same condition. We therefore averaged the responses from 2 s before the stimulus onset (event or choice) to 20 s after its end, for both ER and NER conditions. The three ER scenarios (ER1, ER2, and ER3) and the three NER scenarios (NER1, NER2, and NER3) were combined within the ER and NER conditions, respectively, to increase statistical power and focus on the general effect of emotional relevance. For the statistical analyses, only responses elicited by the event stimuli were considered, and the peak response amplitude was extracted for each participant, ROI, chromophore, and condition as a measure of activation, and paired t-tests were used to assess differences among conditions. To account for multiple comparisons, the resulting p-values were corrected using the false discovery rate (FDR) method proposed by Benjamini and Hochberg.^19^ Responses evoked by the action stimuli were not included in the statistical analysis due to the intrinsic limitation of the block-averaging approach in the presence of temporally overlapping HRs, which is the case for the action blocks as they are followed event blocks without an intervening rest period.

#### 2.5.2 Canonical GLM

The *spm_hrf()* function with all parameters set to default values (Table 3) was used to generate the HRF with the canonical double-gamma model in the SPM framework. This function models both the main peak of the hemodynamic response and the subsequent undershoot, providing an approximation of the expected response in standard experimental paradigms and populations.

**Table 3:** Parameters of the canonical double-gamma model in the SPM framework.

Parameter	Default value (s)
Delay of response (relative to onset)	6
Delay of undershoot (relative to onset)	16
Dispersion of response	1
Dispersion of undershoot	1
Ratio of response to undershoot	6
Onset (s)	0

The design matrix used in our analysis consisted of nine regressors, constructed as follows:

1. Setup scene: modeled the initial setup period at the beginning of each experimental trial.
2. Inter-trial periods.
3. Emotionally relevant event blocks: modeled as a single regressor combining three types of ER scenarios (ER1, ER2, and ER3 events).
4. Nonemotionally relevant event blocks: modeled as a single regressor combining three types of NER scenarios (NER1, NER2, and NER3 events).
5. Emotionally relevant choice blocks: modeled participants’ responses to ER choice. The three ER scenarios (ER1, ER2, and ER3 choices) were combined in a single regressor.
6. Nonemotionally relevant choice blocks: modeled participants’ responses to NER choice. The three ER scenarios (NER1, NER2, and NER3 choices) were combined in a single regressor.
7. Accelerometer data: derived from the squared sum of the raw accelerometer signal across the three axes ($X^{2}+Y^{2}+Z^{2}$), used to account for motion-related artifacts.
8. Short-channel data: average of the data from the two SCC, included to regress out superficial physiological signals.
9. Intercept: modeled the baseline offset for each time series.

The duration of the block in the task-related regressors (1 to 6) was defined based on trigger event markers recorded during the experiment. The GLM was fit separately for each participant, chromophore, and ROI. Beta coefficients were extracted from the model as estimates of task-related activation. For the statistical comparisons, we computed the ER versus NER contrast, separately for the events and choices blocks. Paired t-tests were then used on the resulting contrast maps to identify significant activation patterns across participants. To account for multiple comparisons, the resulting p-values were corrected using the FDR correction method, as before.

#### 2.5.3 AICopt-based GLM

**Fig. 4:**
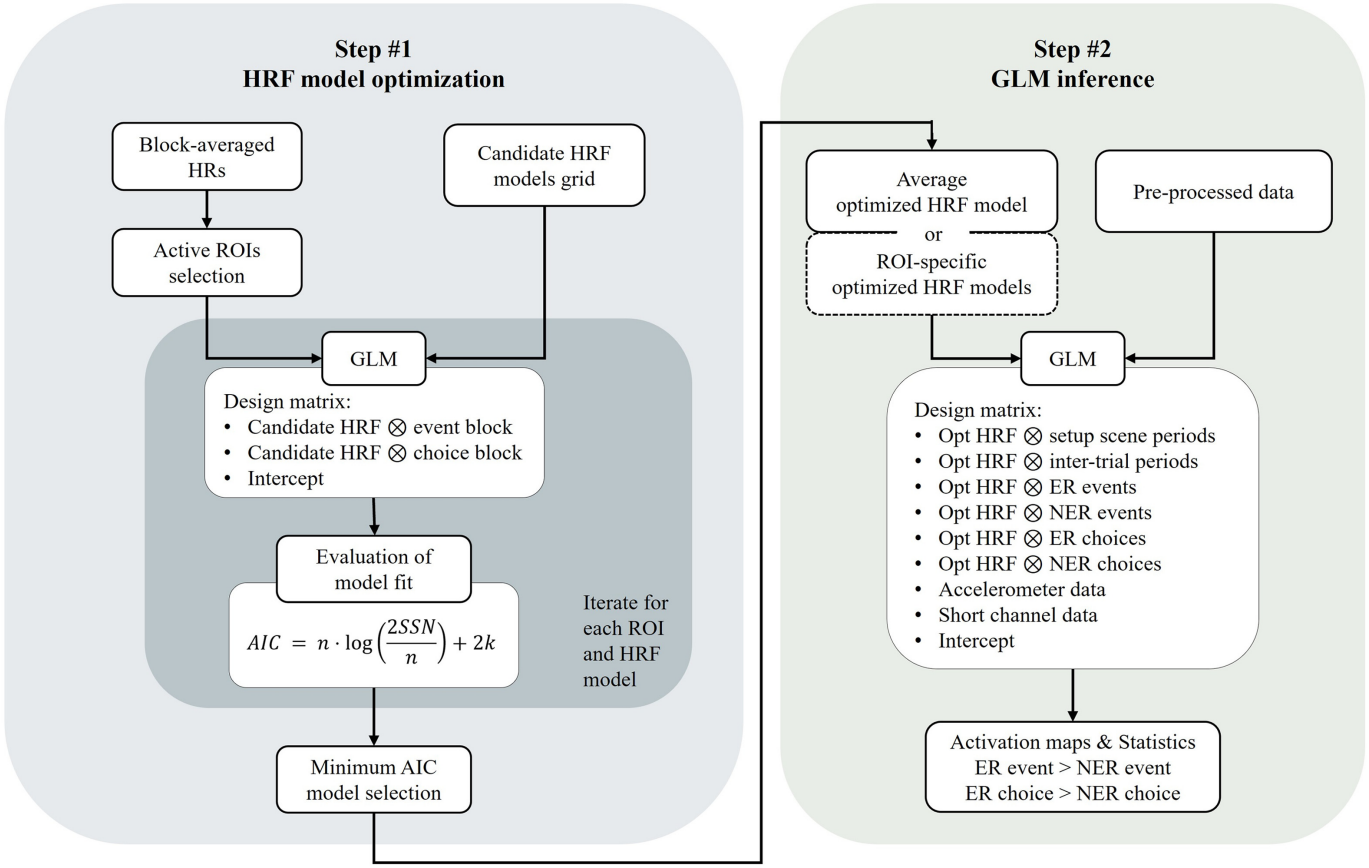
Schematic summary of the main steps of the AICopt workflow.

This approach is structured in two steps (see Fig. 4): the first, to estimate the optimal HRF model from the data via iterative model selection; and the second, to assess its impact on inference using the optimized HRF in a GLM analysis analogous to method (ii). As a first step, to obtain an initial HR estimate for each participant, ROI, and condition, the block averaging used in method (i) was applied to the fNIRS data. To increase the specificity of the following analysis, a subset of ROIs that showed consistent and significant activation following ER events in both HbO and HHb was selected, ensuring that the HRF optimization focused on brain regions likely to exhibit meaningful task-related responses. Multiple GLMs were fitted to the obtained subset of block-averaged HRs, each assuming a different variation of the double-gamma HRF model from SPM. To reduce the method complexity and computational time, we decided to optimize just two of the six parameters in Table 3. The response delay and dispersion were selected as the most impacting parameters. The response delay was varied between 4 and 25 s, with 1 s steps; the response dispersion was varied between 1 and 4, with 0.2 s steps. All possible combinations of the two parameters were tested, and the undershoot dispersion was always set equal to the response dispersion. Finally, the undershoot delay was changed with respect to the canonical model and fixed to 5 s from the onset to match the dataset characteristics. Hereafter, a design matrix composed of three regressors was built:

1. A regressor for the event period (regardless of whether it was ER or NER, as both shared the same timing),
2. A regressor for the choice period (independently of the emotional valence),
3. An intercept term.

The durations of the event and choice blocks were fixed at 8 s as this was the minimum amount of time between an event or choice and the subsequent event in the experimental protocol. At each iteration, the first two regressors were convolved with the HRF model being tested. The same HRF model was applied to both event and choice regressors at each iteration, allowing for a single HRF optimization per condition while accounting for the timing of both stimuli. This iterative GLM fit explicitly accounts for the expected overlap between event- and choice-related responses present in the block-averaged data. Each model fit was then evaluated and compared for the AIC calculated as

$$\mathrm{AIC}=n\cdot \log(\frac{2\mathrm{SSN}}{n})+2k,$$ (1)

where SSN is the sum of squared errors, n is the number of observations, and k is the number of model parameters.^13^ The HRF model associated with the model showing the minimum average AIC over all participants was then selected for each Hb species, ROI, and condition. Subsequently, we applied the GLM in two ways:

1. Individually optimized HRFs: for each ROI, we used its own optimized HRF model, derived through the AICopt procedure. For those ROIs that did not show significant activation in the block-averaging step, we used the average HRF model computed across all fitted regions.
2. Averaged HRF model: a single, averaged HRF model across the selected ROIs was computed and used to examine the performance of a generalized HRF model.

Both methods were performed to investigate whether it was necessary to perform a region-specific analysis or whether it was sufficient to use the optimized model averaged across regions.

Hereafter, the same design matrix as in the Canonical GLM approach (ii) was used, ensuring that any observed differences in results could be attributed to the assumed HRF model rather than to differences in experimental design modeling. The statistical test for the ER versus NER contrast was also performed following the same procedure as in method (ii).

### 2.6 Statistical Comparison of HRF Models’ Performance

For comparing results obtained using the GLM approach while assuming different HRF models, the AICopt-based GLM using the averaged HRF model (hereafter AICopt_avg) was taken as the reference, and two comparisons were performed: (i) AICopt_avg versus the canonical adult GLM, to assess whether a data-driven preschooler HRF provides superior sensitivity relative to the standard adult-based approach; and (ii) AICopt_avg versus the AICopt-based GLM using ROI-specific optimized HRFs (hereafter AICopt_ROIs), to assess whether region-level optimization provides additional benefit over a single group-level, averaged model. In both cases, two levels of analysis were performed on the beta coefficients extracted for the ER > NER contrast. First, a linear mixed-effects model (LME) was fitted separately for each combination of chromophore (${\mathrm{HbO}}_{2}$, HHb) and contrast (ER > NER event, ER > NER choice), yielding four models per comparison. In each LME, the beta coefficient for the considered contrast was the dependent variable, whereas the HRF model (AICopt_avg and the compared model) was entered as a fixed effect. Participant and ROI were included as crossed random intercepts to account simultaneously for inter-individual variability in overall response amplitude and for region-specific differences. This method yields a global main effect of the HRF model that pools evidence across all ROIs and participants simultaneously. Second, to characterize the regional distribution of sensitivity differences, ROI-level pairwise comparisons were performed using two-sided paired t-tests on the beta coefficients, with effect sizes quantified using Cohen’s d (computed as the mean of the pairwise differences divided by their standard deviation).

## 3 Results

### 3.1 Block Averaging

**Fig. 5:**
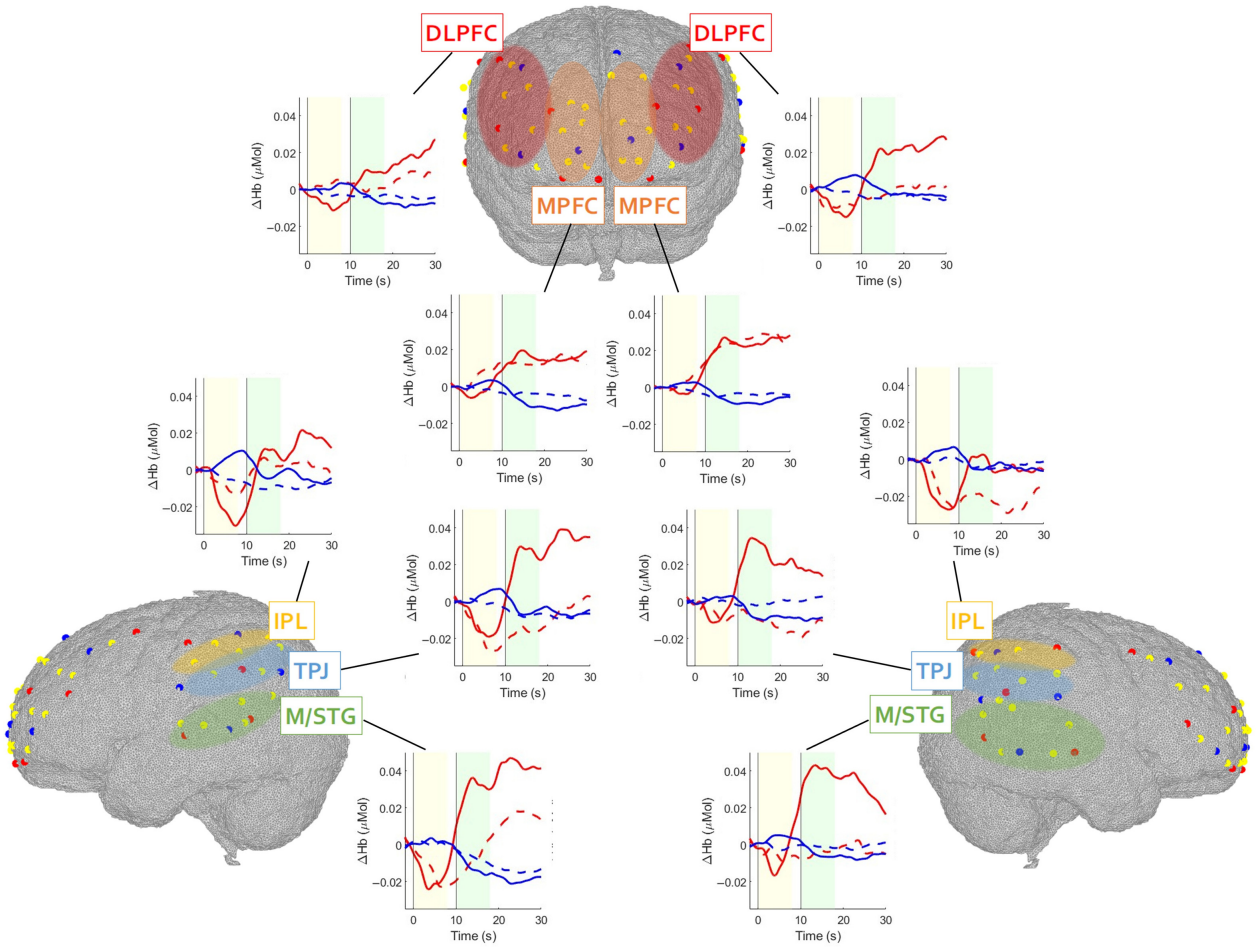
Group-averaged HRs for ${\mathrm{HbO}}_{2}$ (in red) and HHb (in blue), comparing the ER condition (solid line) with the NER condition (dotted line) between 2 s before the event stimuli (yellow shaded area) onset and 20 s past the action block (green shaded area) start.

Figure 5 displays the group-averaged HRs obtained using the block-averaging pipeline for both ${\mathrm{HbO}}_{2}$ and HHb, comparing the ER condition with the NER condition between 2 s before the event stimuli onset and 20 s past the action block start. From the figure, it is clear that the shape and timing of the HR deviate substantially from the canonical HRF typically assumed in adult-based GLM analyses. Although the canonical model has a peak amplitude around 5 s after stimulus onset,^7^ in our dataset, the peak response was consistently observed between 10 and 20 s post-onset across all ROIs. In addition, a pronounced undershoot around 5 s was visible for most ROIs.

**Fig. 6:**
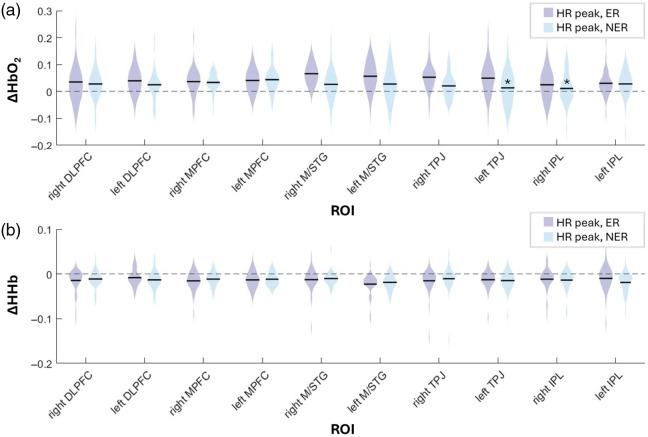
Distribution of peak response amplitudes for both ${\mathrm{HbO}}_{2}$ (a) and HHb (b) across ROIs. Distributions marked with an asterisk are the ones for which the null hypothesis was not rejected.

**Fig. 7:**
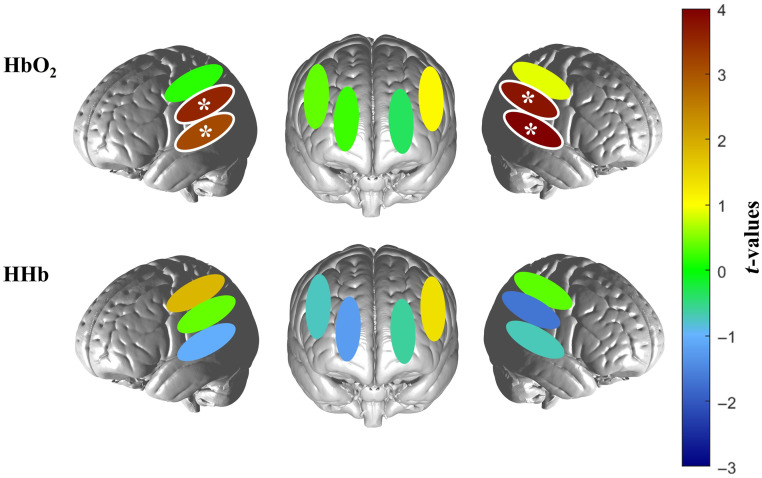
T-value maps derived for the ER event versus NER event contrast using the block averaging pipeline. Positive t-values indicate increased concentration for both ${\mathrm{HbO}}_{2}$ and HHb. The expected task-related activation consists of increased ${\mathrm{HbO}}_{2}$ and decreased HHb concentrations. Regions showing uncorrected statistical significance are outlined with a white contour, whereas those surviving FDR correction for multiple comparisons are marked with an asterisk.

To identify those ROIs that showed task-related activation, we extracted the peak response amplitude in the 10 to 20 s window following event block start for both ${\mathrm{HbO}}_{2}$ and HHb signals, across all ROIs and participants. We then tested the null hypothesis that this peak did not differ significantly from baseline. The results revealed that all ROIs showed significant activation in response to both ER and NER events for both chromophores (the only exceptions were left TPJ and right IPL, which did not significantly differ from zero in the NER condition in the ${\mathrm{HbO}}_{2}$ signal only). See Fig. 6 for the distribution of peak response amplitudes for both ${\mathrm{HbO}}_{2}$ and HHb across all ROIs. The t-value maps derived for the ER versus NER events contrast are depicted in Fig. 7, for both ${\mathrm{HbO}}_{2}$ and HHb. For ${\mathrm{HbO}}_{2}$, four ROIs showed significant activation after FDR correction: bilateral TPJ and bilateral M/STG (no ROIs showed significant activations for HHb). We did not estimate the activation map for the ER versus NER choice contrast due to the intrinsic limitation of the block-averaging method, which is unsuitable in the presence of overlapping HRs.

### 3.2 Canonical GLM

**Fig. 8:**
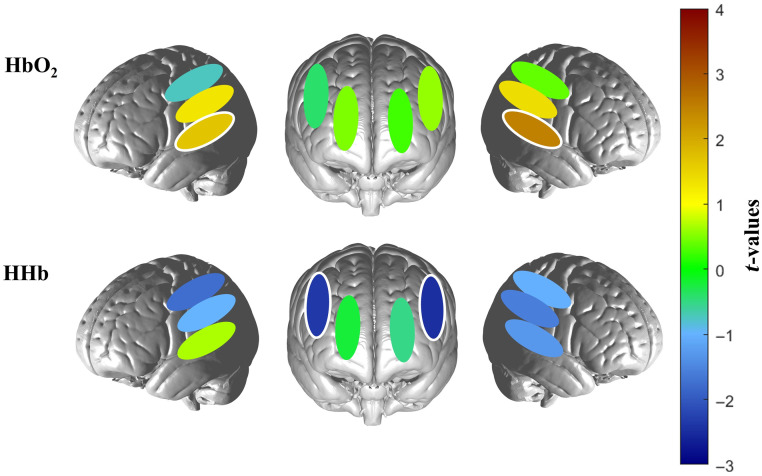
T-value maps derived for the ER event versus NER event contrast using the canonical GLM pipeline. Positive t-values indicate increased concentration for both ${\mathrm{HbO}}_{2}$ and HHb. The expected task-related activation consists of increased ${\mathrm{HbO}}_{2}$ and decreased HHb concentrations. Regions showing uncorrected statistical significance are outlined with a white contour.

We first compared the activations for ER versus NER event (Fig. 8), and we found that only left and right M/STG showed greater activation for ER compared with NER in ${\mathrm{HbO}}_{2}$, and left and right DLPFC showed greater activation for ER compared to NER in HHb. However, none of these survived FDR correction for multiple comparisons.

**Fig. 9:**
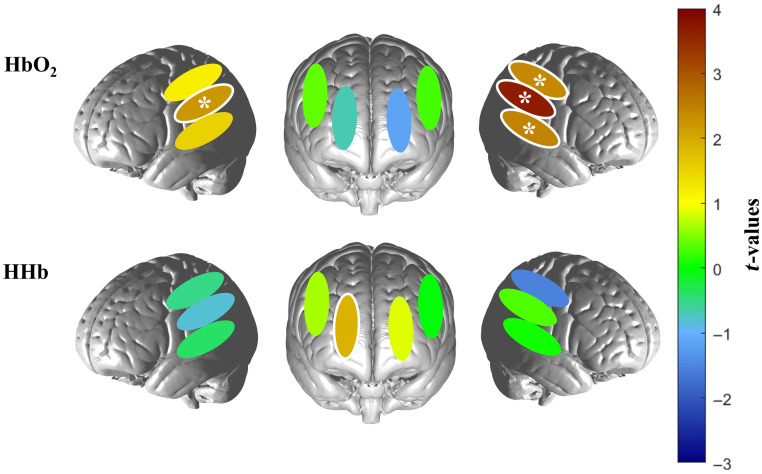
T-values maps derived for the ER choice versus NER choice contrast using the canonical GLM pipeline. Positive t-values indicate increased concentration for both ${\mathrm{HbO}}_{2}$ and HHb. The expected task-related activation consists of increased ${\mathrm{HbO}}_{2}$ and decreased HHb concentrations. Regions showing uncorrected statistical significance are outlined with a white contour, whereas those surviving FDR correction for multiple comparisons are marked with an asterisk.

For the ER versus NER choice contrast (Fig. 9), right IPL and M/STG, and bilateral TPJ showed significantly greater activation for ER choice compared with NER choice in the ${\mathrm{HbO}}_{2}$ maps, which survived FDR correction. However, in the HHb maps, there were no significant differences in activations between ER and NER choice that survived FDR correction.

### 3.3 AICopt-based GLM

**Fig. 10:**
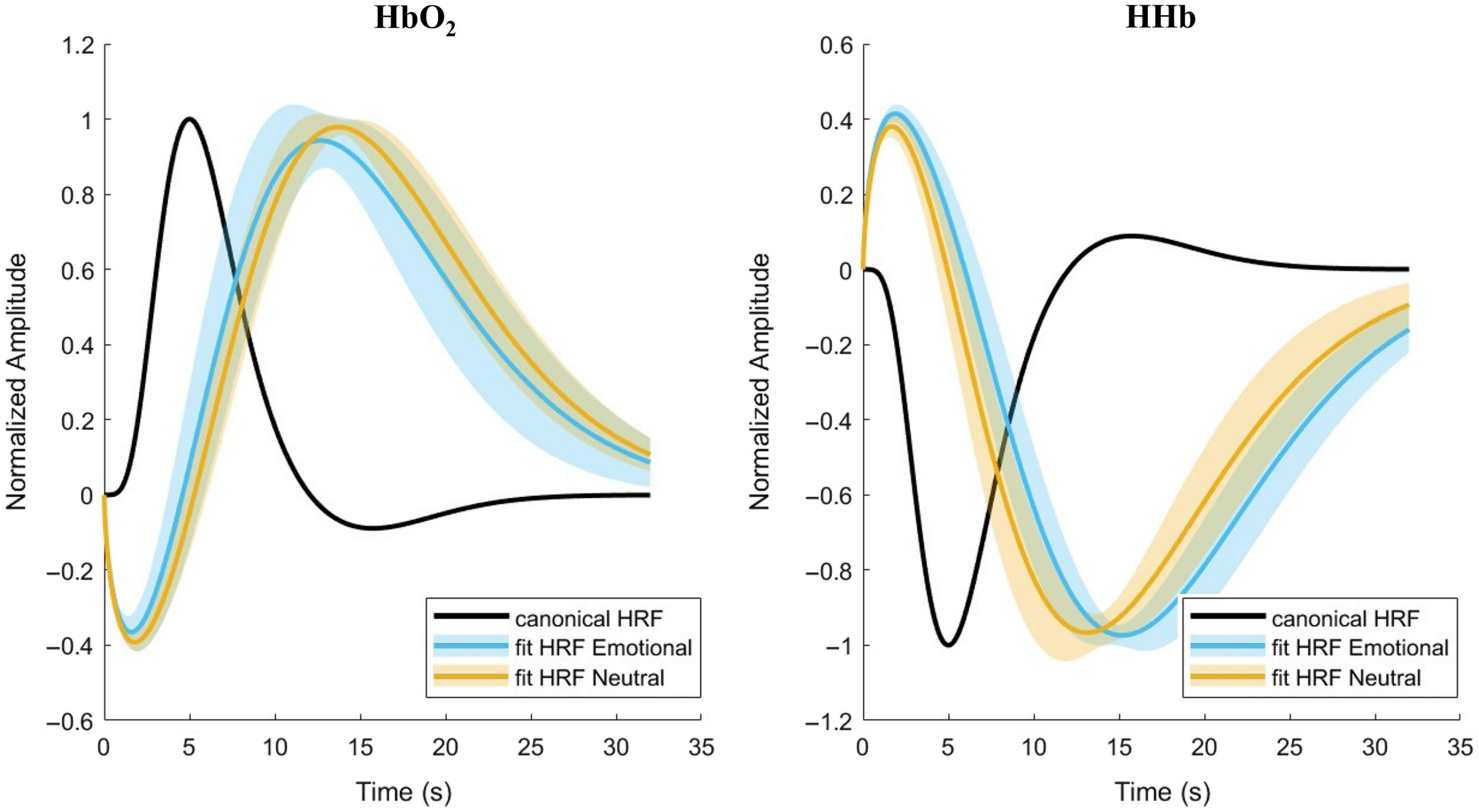
Average HRF models estimated using the AICopt method for the ER (in blue) and NER (in yellow) conditions. The canonical HRF model is also shown in black. The shaded areas around the average curves represent the standard deviation across the considered ROIs.

Figure 10 presents the average HRF models estimated using the AICopt method for the selected ROIs that previously showed consistent task-related activation in the block-averaging analysis (Sec. 3.1, Fig. 6). All ROIs were included in the HRF optimization for the ER condition, whereas left TPJ and right IPL were excluded for the NER condition. Figure 10 shows the optimized HRFs separately for the ER and NER conditions, for both ${\mathrm{HbO}}_{2}$ and HHb, compared with the canonical HRF. Notably, although the optimized HRFs deviate from the canonical shape exhibiting delayed peaks and different dispersion characteristics, the differences between the ER and NER conditions are relatively small (i.e., the response peaks differ <2  s on average, whereas the response dispersion is the same), suggesting comparable temporal dynamics across the two conditions. To provide a quantitative characterization of the optimized HRFs, Table 4 reports the mean and standard deviation of the fitted HRF parameters across subjects for each ROI, as well as their average and standard deviation across ROIs, which were used to derive the group-level HRFs models shown in Fig. 10. The table shows that, although inter-subject variability in the estimated parameters is relatively high, the average parameter values are highly consistent across ROIs.

**Table 4:** Mean and standard deviation of the optimized parameters (response delay and response dispersion) estimated using the AICopt method. Values are reported across subjects for each ROI (r- for right, l- for left), hemoglobin species (${\mathrm{HbO}}_{2}$ or HHb), and experimental condition (ER or NER). The grand average across ROIs, to obtain the group-level HRF models, is also reported (last row).

ROI	Response delay				Response dispersion			
	${\mathrm{HbO}}_{2}$		HHb		${\mathrm{HbO}}_{2}$		HHb	
	ER	NER	ER	NER	ER	NER	ER	NER
rDLPFC	11.3±5.1	12.9±5.7	10.9±5.2	11.4±5.6	2.3±0.9	1.9±0.9	2.1±0.9	2.1±0.9
lDLPFC	11.6±5.0	11.9±6.0	13.6±5.2	12.7±4.9	2.3±0.9	2.2±0.9	2.4±0.8	2.4±0.9
rMPFC	11.4±5.6	13.2±5.1	13.0±5.6	13.5±5.5	2.5±0.8	2.0±1.0	2.5±0.8	2.1±0.9
lMPFC	11.5±4.8	11.8±4.9	14.2±4.8	12.4±5.0	2.3±0.9	2.3±0.9	2.4±0.9	2.2±1.0
rM/STG	12.4±4.7	11.0±5.7	10.8±5.9	9.9±4.5	2.2±1.0	2.4±0.9	2.3±0.9	2.1±1.0
lM/STG	11.9±5.2	11.5±5.4	12.6±5.5	12.8±5.0	2.5±0.8	2.4±0.9	2.5±0.8	2.0±1.0
rTPJ	12.0±5.2	12.8±6.1	11.9±5.2	13.5±4.8	2.1±0.9	2.3±0.8	2.1±0.9	2.1±1.0
lTPJ	12.5±5.6	11.9±5.3	11.6±6.0	11.6±5.0	2.3±0.9	2.1±0.9	2.1±0.9	2.0±1.0
rIPL	11.9±5.6	12.1±5.5	10.5±5.3	11.2±4.4	2.1±0.9	2.3±0.9	2.0±1.0	2.0±1.0
lIPL	12.3±5.7	10.5±5.8	10.8±5.3	9.8±5.5	2.1±0.9	2.0±0.9	2.3±0.9	2.2±1.0
Average	11.9±0.4	12.0±0.8	12.0±1.3	11.9±1.3	2.3±0.1	2.2±0.2	2.3±0.2	2.1±0.1

**Fig. 11:**
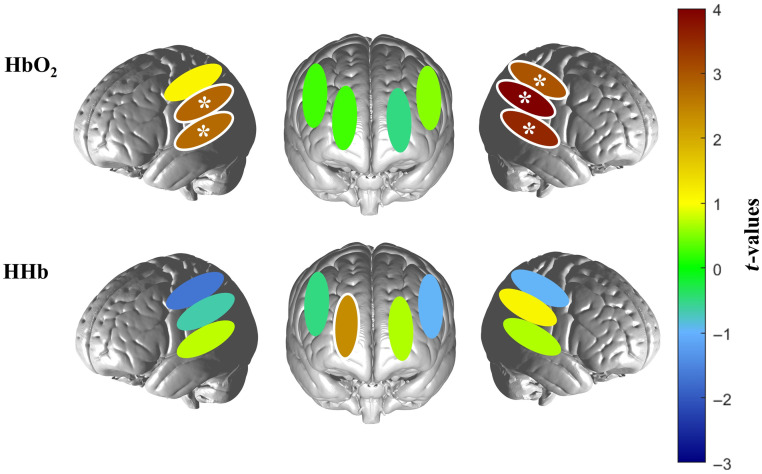
T-value maps derived for the ER event versus NER event contrast using the AICopt-based GLM pipeline. Positive t-values indicate increased concentration for both ${\mathrm{HbO}}_{2}$ and HHb. The expected task-related activation consists of increased ${\mathrm{HbO}}_{2}$ and decreased HHb concentrations. Regions showing uncorrected statistical significance are outlined with a white contour, whereas those surviving FDR correction for multiple comparisons are marked with an asterisk.

**Fig. 12:**
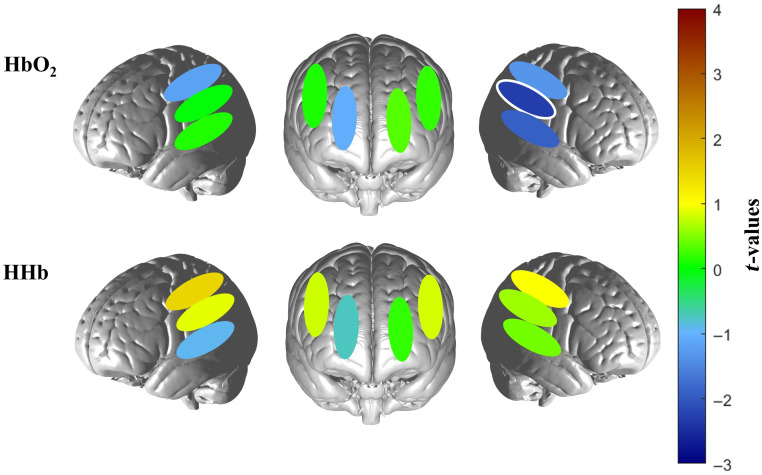
T-value maps derived for the ER choice versus NER choice contrast using the AICopt-based GLM pipeline. Positive t-values indicate increased concentration for both ${\mathrm{HbO}}_{2}$ and HHb. The expected task-related activation consists of increased ${\mathrm{HbO}}_{2}$ and decreased HHb concentrations. Regions showing uncorrected statistical significance are outlined with a white contour.

Given the high similarity between results obtained with AICopt_ROIs and those derived from AICopt_avg, for simplicity, we chose to report activation maps for the GLM analysis using AICopt_avg only. The resulting t-value maps are shown in Figs. 11 and 12. Bilateral M/STG and TPJ, and right IPL showed significantly greater activation for ER compared to NER events in the HbO map. Only the right TPJ showed a significantly greater activation for NER compared to ER in the HHb maps, but these results did not survive FDR correction for multiple comparisons.

### 3.4 Statistical Comparison of HRF Models’ Performance

To test whether AICopt_avg provided a statistically significant overall improvement in sensitivity relative to each compared model, the LME analysis described in Sec. 2.6 was applied. The results are summarized in Table 5. For the comparison between AICopt_avg and the canonical adult HRF, a significant global main effect of HRF type was found for the event contrast in both chromophores: ${\mathrm{HbO}}_{2}$ and HHb, indicating that AICopt_avg yields significantly larger contrast estimates than the canonical HRF across all ROIs and participants simultaneously. For the choice contrast, the LME revealed significantly larger betas under the canonical HRF for ${\mathrm{HbO}}_{2}$, whereas no significant overall difference was found for HHb. As discussed below, the positive estimate for ${\mathrm{HbO}}_{2}$-choice is interpreted as a global quantification of the misattribution effect rather than evidence of canonical superiority. For the comparison between AICopt_avg and AICopt_ROIs, the LME revealed no significant differences across any of the four conditions, indicating that ROI-level optimization provides no significant additional benefit over the averaged model.

**Table 5:** Summary of LME results for the HRF-model main effect. Negative (positive) estimates indicate AICopt_avg yields larger (smaller) beta coefficients than the alternative model. CI = confidence interval.

Comparison	Contrast	Estimate	95% CI	p value
Canonical adult versus AICopt_avg	$\left({\mathrm{HbO}}_{2};\text{event}\right)$	−0.0093	[−0.0145, −0.0041]	<0.001*
	(HHb; event)	−0.0031	[−0.0050, −0.0012]	0.002*
	$\left({\mathrm{HbO}}_{2};\text{choice}\right)$	+0.0124	[+0.0069,+0.0178]	<0.001*
	(HHb; choice)	−0.0015	[−0.0034, +0.0004]	0.125
AICopt_ROIs versus AICopt_avg	$\left({\mathrm{HbO}}_{2};\text{event}\right)$	+0.0002	[−0.0051, +0.0054]	0.950
	(HHb; event)	−0.0006	[−0.0026, +0.0015]	0.598
	$\left({\mathrm{HbO}}_{2};\text{choice}\right)$	−0.0004	[−0.0047, +0.0039]	0.867
	(HHb; choice)	+0.0002	[−0.0016, +0.0020]	0.850

**Fig. 13:**
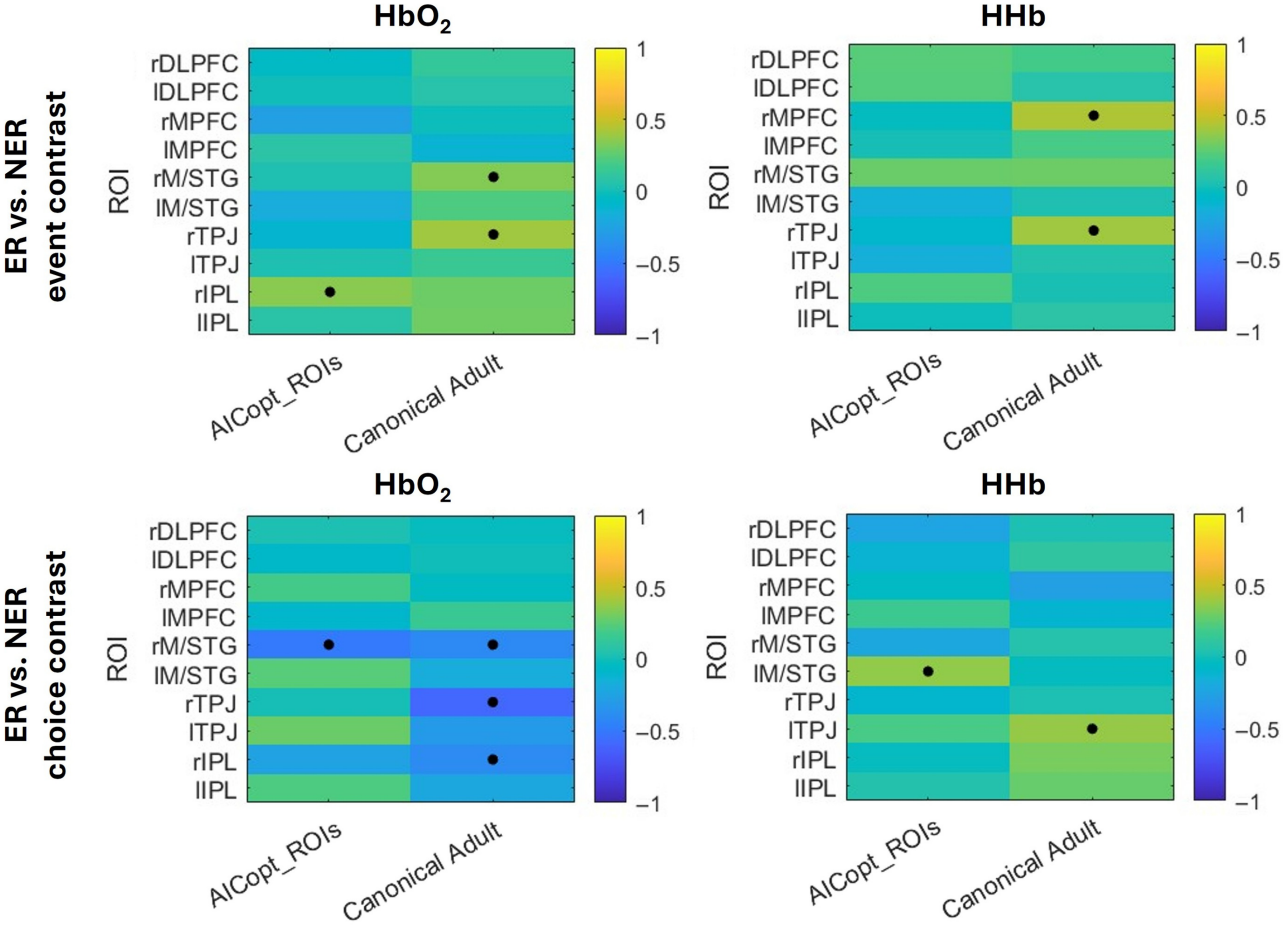
Heatmaps of effect sizes for the ER versus NER event contrast (a) and the ER versus NER choice contrast (b) across ROIs. Each column compares the AICopt_avg against one alternative model: left column, AICopt_avg versus AICopt_ROIs; right column, AICopt_avg versus canonical adult HRF. Cohen’s d is computed as the alternative model minus AICopt_avg: negative values (cooler colors) indicate ROIs where the AICopt_avg yields larger contrast estimates (i.e., greater sensitivity); positive values indicate ROIs where the compared model yields larger estimates. Black dots denote statistical significance (p<0.05, using paired t-test).

To characterize the regional distribution of these sensitivity differences, ROI-level pairwise was performed on the beta coefficients for the ER versus NER event and ER versus NER choice contrasts. The results are summarized in Fig. 13, which reports heatmaps of effect sizes (Cohen’s d, computed as the alternative model: canonical adult or AICopt_ROIs, minus AICopt_avg) across ROIs: negative values (cooler colors) indicate ROIs where AICopt yields larger contrast estimates than the compared model, positive values (warmer colors) indicate the reverse, and black dots mark ROIs reaching statistical significance (p<0.05) on the individual paired test. The comparison shows that effect sizes obtained with AICopt_ROIs are largely comparable to those obtained using the AICopt-derived HRF averaged across ROIs, with only a few ROIs showing notable differences. By contrast, clearer differences emerge when comparing the AICopt-based HRF with the canonical adult HRF. In particular, for the event contrast, regions that did not show significant activation with the canonical GLM but did with the AICopt-based GLM (e.g., right M/STG and TPJ in ${\mathrm{HbO}}_{2}$) exhibited significantly larger effect sizes with the AICopt HRF. Conversely, ROIs showing significant effects for the choice contrast only with the canonical GLM displayed smaller effects when using the AICopt-based HRFs (d<0).

## 4 Discussion

In this study, we evaluate a new method (AICopt) designed to advance fNIRS data analysis in nonconventional settings, where overlapping hemodynamic responses are common and difficult to recover with conventional approaches. Our dataset was acquired in a naturalistic, immersive VR environment, on 3-to-5-year-old children. We evaluated how our AICopt method performed in comparison with two conventional analysis methods: block averaging and canonical GLM.

The block-averaging approach, typically used in developmental studies on children due to expected deviations in HR shape from adult-based canonical responses, revealed clear hemodynamic responses across most ROIs. From the group-level analysis, the average HRs showed remarkable deviation from the canonical HRF, with peak amplitudes observed 10 to 20 s post-stimulus onset, compared with the 5-s delay expected by the canonical model. An undershoot with a peak around 5 s was also visible across most ROIs. Although this could partly reflect a residual carry-over effect from preceding stimuli, similar effects have been previously reported in developmental fNIRS studies employing well-structured block designs,^20^^,^^21^ suggesting that it may also reflect characteristics of the hemodynamic response in young populations. These findings confirm that the canonical HRF may be inappropriate for analyzing our dataset as it does not accurately reflect the temporal dynamics of the measured HRs. Using the maximum response amplitude between 10 and 20 s post-event-block-onset, we found significant task-related activations in almost all ROIs for both ER and NER conditions, with only a few exceptions for ${\mathrm{HbO}}_{2}$ in the NER condition. Bilateral M/STG and TPJ in the ${\mathrm{HbO}}_{2}$ map showed greater activation for the ER compared with the NER event. However, we were not able to explore whether there were any differences in activations between the ER and the NER choice. In fact, the block-averaging method assumes that the response to each stimulus is independent and does not account for potential overlap between responses to different stimuli. In our protocol, every choice occurred immediately after its preceding event, with no intervening baseline, so that choice-evoked responses were partially superimposed to the event responses. Consequently, the baseline-correction step in the block averaging pipeline cannot reliably recover the HRs.

The results obtained using the canonical GLM approach may reveal limited sensitivity in detecting condition-specific activations, particularly for the event contrast. In fact, only a few ROIs (i.e., left and right M/STG for ${\mathrm{HbO}}_{2}$, and left and right DLPFC for HHb) exhibited significantly greater activation for ER compared with the NER event prior to multiple comparison correction, and none survived FDR correction. Conversely, several ROIs showed greater activation for the ER compared with the NER choice and survived the FDR correction. When considered alongside the results recovered with the block-averaging method, these findings suggest a potential misattribution of neural responses in the canonical GLM analysis. Specifically, the block-averaged responses showed robust hemodynamic changes following the ER events, with clear peaks and undershoots that were consistent across conditions and ROIs. In the GLM method, due to the temporal overlap of stimuli (events and choices) and the fixed HRF assumption typical of the GLM method, it seems that some of the event-related HRs were erroneously linked to the choice stimuli rather than the first event. These results highlight the limitations of applying standard adult-based models in developmental neuroimaging, demonstrating that using an inappropriate HRF can lead to nonnegligible errors in data interpretation.

Using the AICopt method for HR model optimization, we extracted a set of data-driven HRFs that showed consistent temporal dynamics across ROIs and conditions, indicating the robustness of this approach. In particular, the analysis of the fitted HRF parameters revealed that, although inter-participant variability in optimized response delay and dispersion was relatively high, the average parameter values were remarkably homogeneous across ROIs, supporting the use of a single optimized HRF model at the group level. Future studies on larger cohorts will be necessary to determine whether inter-individual variability in HRF parameters relates to age or sex. Notably, preliminary analyses in which the initial negative undershoot observed in the block-averaging HRs was either freely fitted or ignored resulted in unstable or suboptimal model fits, whereas explicitly fixing it improved model stability and convergence, suggesting that this feature could not be fully explained as an artificial carry-over effect from preceding stimuli. After the subsequent AICopt-based GLM analysis, we obtained results that largely overlapped the ones observed for the block averaging method for the event. In fact, the bilateral M/STG and TPJ showed greater activation for the ER compared with the NER events for both the block averaging and AICopt method. The AICopt method also showed greater activation of the right IPL for the ER compared with the NER events. The AICopt method did not identify any significant difference in activation that survived FDR correction between ER and NER choice. Overall, our findings using the AICopt-based GLM are more consistent with what observed using the block averaging method compared with the canonical GLM. Notably, block averaging may give more faithful results because it operates directly on the measured time courses, avoiding any HRF assumption, whereas GLM-based inferences are sensitive to a priori HRF modeling and are prone to bias errors when the true HRF deviates from the canonical shape (which we expect in our dataset). In addition, these results better aligned with expectations: the hemodynamic response to choice stimuli was less pronounced than the response to the event stimuli. This might be due to the experimental structure, where choice periods follow closely after the events and are expected to elicit a smaller vascular response due to partial saturation.^22^ The canonical GLM approach, however, revealed stronger activations for the choice condition, particularly in ${\mathrm{HbO}}_{2}$. This discrepancy is likely due to the mismatch between the canonical HRF and the actual temporal dynamics of the measured HRs. Crucially, the AICopt method offers advantages over block averaging as well, enabling the use of GLM analysis in the presence of overlapping events, thus allowing us to test both event- and choice-evoked HRs despite their temporal proximity. Taken together, the results presented in this study suggest the proposed method as a possible solution to a longstanding methodological trade-off in the fNIRS literature: block-averaging approaches are robust in developmental populations because they avoid a priori HRF assumptions, but they are limited to protocols where fast stimulus series could lead to overlapped responses, whereas canonical GLM analysis can handle fast-paced designs but may fail when the assumed HRF does not match the true characteristics of the measured responses. The proposed AICopt method addresses both limitations within a single framework, by retaining the ability of GLM analyses to disentangle overlapping responses while replacing fixed HRF assumptions with a data-driven optimization tailored to the specific dataset under analysis.

Our results provide direct statistical support for our hypotheses that using an optimized, data-driven HRF when applying the GLM analysis on data from developmental populations and from a more realistic set-up can significantly improve the sensitivity of the obtained activation maps. This improvement is especially relevant in studies involving children and nonstandard experimental paradigms, where significant deviations from the canonical HR are expected. The LME analysis revealed a significant global main effect of HRF type for the event contrast, in favor of AICopt_avg over the canonical adult HRF, for both ${\mathrm{HbO}}_{2}$ and HHb. The magnitude of this global effect is modest in absolute terms, but its statistical robustness across ROIs and participants proves its reliability. The proposed AICopt approach, by optimizing the HRF shape from the data itself, offers a robust framework for increasing the sensitivity to task-related responses by accounting for temporal variability in the HR and reducing the risk of responses misattribution. The LME results for the choice contrast require careful interpretation. The canonical HRF yielded significantly larger ${\mathrm{HbO}}_{2}$ betas for the ER > NER choice contrast than AICopt_avg. This might appear to indicate superior sensitivity of the canonical model for choice-related responses; however, this finding must be read together with the block-averaging results, which showed that the true preschooler hemodynamic response peaks at around 10 to 20 s post-stimulus. Because the canonical HRF peaks at ∼5  s, the canonical GLM likely misaligns the event regressor with respect to the true neural response, causing a portion of the event-evoked activation to be incorrectly absorbed by the choice regressor. Therefore, the LME result quantifies this misattribution at the global level, indicating that the canonical model inflates choice-related betas relative to AICopt_avg across all ROIs. The absence of a significant difference for HHb-choice is consistent with the generally weaker and noisier HHb signal, which likely reduces the misattribution effect below detectable levels. Finally, the LME comparison between AICopt_avg and AICopt_ROIs yielded no significant differences across any condition. Although this comparison is limited by the sample size and the small expected differences between the two models, the negligible effect sizes suggest that ROI-specific HRF optimization offers no additional benefit for this dataset and that AICopt_avg represents a simple and sufficient choice for future applications.

Given the growing adoption of fNIRS in naturalistic, clinical, and other nontraditional settings, as well as across more diverse populations, the proposed AICopt method provides a practical data-analysis workflow that researchers in the field can use to obtain more accurate and interpretable GLM results by tuning the analysis to their own datasets. A wider use of data-driven HRF optimization methods will indeed improve inference in experimental settings involving events in close temporal proximity and populations for which the canonical HRF may be not appropriate.

## 5 Conclusion

Our results suggest that data-driven HRF modeling represents a valuable step when analyzing fNIRS data from atypical populations such as young children, particularly in studies employing naturalistic or weakly structured paradigms, where the use of standard protocol designs is likely unsuitable and overlapping hemodynamic responses are often unavoidable. The proposed AICopt method, through its data-driven optimization of the HRF, offers a promising solution to adapt the standard GLM analysis approach to unconventional datasets. The AICopt framework addresses two complementary and longstanding methodological challenges in the fNIRS literature. On the one hand, canonical GLM approaches based on adult HRF models may perform poorly on developmental data, potentially leading to misattribution of neural responses. On the other hand, block-averaging methods, although robust due to their model-free nature, are limited to protocols with sufficiently spaced events and cannot reliably handle stimulation following close in time. By combining data-driven HRF optimization with a GLM framework, the proposed method enables the analysis of closely spaced events while avoiding strong a priori assumptions about HRF dynamics. In this study, the proposed method proved capable of revealing more reliable activation patterns that matched those identified by the block averaging approach. Moreover, it effectively distinguished between overlapping responses and aligned results with what was expected based on the used task structure, detecting stronger responses to events compared with choice-related activations.

Overall, our study stands for carefully considering HRF modeling when applying GLM analyses to developmental fNIRS data acquired in nonstandard experimental settings. Although block averaging remains appropriate for well-controlled protocols when explicitly designed to accommodate slower hemodynamics typical of young populations, data-driven HRF optimization offers a robust and flexible strategy to improve confidence in the estimated task-related activations that may otherwise be misinterpreted using standard approaches.

## Data Availability

The data that support the findings of this study cannot be publicly shared due to privacy issues but are available from the senior author upon reasonable request.
